# A CNN-LSTM Architecture for Marine Vessel Track Association Using Automatic Identification System (AIS) Data

**DOI:** 10.3390/s23146400

**Published:** 2023-07-14

**Authors:** Md Asif Bin Syed, Imtiaz Ahmed

**Affiliations:** Industrial and Management Systems Engineering Department, West Virginia University, Morgantown, WV 26506, USA; ms00110@mix.wvu.edu

**Keywords:** Maritime Track Association, neural networks, deep learning, automatic identification system (AIS), multi-object tracking

## Abstract

In marine surveillance, distinguishing between normal and anomalous vessel movement patterns is critical for identifying potential threats in a timely manner. Once detected, it is important to monitor and track these vessels until a necessary intervention occurs. To achieve this, track association algorithms are used, which take sequential observations comprising the geological and motion parameters of the vessels and associate them with respective vessels. The spatial and temporal variations inherent in these sequential observations make the association task challenging for traditional multi-object tracking algorithms. Additionally, the presence of overlapping tracks and missing data can further complicate the trajectory tracking process. To address these challenges, in this study, we approach this tracking task as a multivariate time series problem and introduce a 1D CNN-LSTM architecture-based framework for track association. This special neural network architecture can capture the spatial patterns as well as the long-term temporal relations that exist among the sequential observations. During the training process, it learns and builds the trajectory for each of these underlying vessels. Once trained, the proposed framework takes the marine vessel’s location and motion data collected through the automatic identification system (AIS) as input and returns the most likely vessel track as output in real-time. To evaluate the performance of our approach, we utilize an AIS dataset containing observations from 327 vessels traveling in a specific geographic region. We measure the performance of our proposed framework using standard performance metrics such as accuracy, precision, recall, and F1 score. When compared with other competitive neural network architectures, our approach demonstrates a superior tracking performance.

## 1. Introduction

Tracking moving objects using both location and temporal information has enormous implications. It has the potential to improve maritime surveillance and security systems, as well as facilitate collision prevention and alert marine vessels to potential hazards. Tracking vessels is related to many active research domains such as track association [1], detection of the anomalous vessels [2,3], trajectory prediction [4,5], clustering the moving objects with similar patterns [6,7], and so on. Among them, track association, the concept of connecting unlabeled moving objects to their actual tracks is comparatively less explored. The unavailability of reliable data, the absence of advanced computational techniques for exploiting big Automatic Identification System (AIS) datasets, the inherent complexity of the trajectory of the marine vessels, and the intrinsic behavior of these vessels can be cited as some of the underlying reasons for this setback.

Spatio-temporal, geo-referenced datasets are rapidly expanding and will continue to do so in the near future due to technological advancements as well as social and commercial factors. The introduction of the AIS, which allows neighboring ships to communicate frequently with their location and navigation status via a radio signal, has enabled researchers to get their hands on datasets rich in spatio-temporal information [8]. AIS data are collected from satellites and ground stations located all over the world. AIS data facilitates the mapping and characterization of maritime human and vessel activities, thus allowing for the real-time geo-tracking and identification of vessels equipped with AIS. Hence, in addition to its initial application in collision avoidance, AIS is now also a massive data source of unparalleled quality for diverse tracking tasks [9].

The AIS dataset contains the location and motion features of the vessels. Each data point or row in the AIS data file is represented by a time-sequenced node that contains the vessel’s coordinates, speed, and traveling direction. Each node also has an associated time stamp indicating the data collection time. The nodes are arranged according to these time stamps. Additionally, each vessel in the AIS data is assigned a unique maritime mobile service identity (MMSI) number. This number helps to associate each node with the respective vessels. If this number is missing or purposefully concealed for a single node or stretch of nodes, a successful tracking algorithm should still be able to associate them to their true tracks. To date, several studies have used AIS datasets and developed algorithms to carry out trajectory prediction, anomaly detection, and clustering. However, most of these studies ignored some of the unique characteristics of these sea-going vessels [1,10,11].

Vessel movement patterns can vary depending on diverse factors such as current location, shipping route, movement of other vessels, environmental factors, etc. Thus, it is no wonder that track association has been a challenging task for decades. Moreover, a ship can suddenly stop transmitting signals for various reasons ranging from unexpected equipment failure to intentionally hiding the true direction. Since a ship could change direction, increase speed significantly, or stop completely during this time, it would be significantly more difficult to correlate nodes to the correct lane after a long period of no communication. To overcome this challenge, it is important to mine the long-term temporal pattern of vessels.

Unlike land vessels, sea vessels do not change their positions and directions abruptly in the open sea and thus making it easier to track them. However, track association becomes more challenging when the vessels are close to a port. In a port, several vessels are docked close together, making it difficult to distinguish between them. Additionally, they frequently adjust their position in order to park or make room for other vessels. Consequently, it requires extra caution to track vessels near port [1]. It is apparent that, for successful tracking, it is important to take into account the vessel’s location and previous movement patterns. In other words, the tracking algorithm should possess the ability to model both the spatial and temporal patterns hidden in the sequential observations.

A variety of techniques have been developed in recent years for tracking moving objects. Multi-object tracking models [12,13,14] such as global nearest neighbor (GNN), joint probabilistic data association (JPDA), and multiple hypothesis tracking (MHT) are some of the most widely used approaches, with Kalman filtering at the core of these algorithms. However, Kalman filtering is more suitable for tracking linear motion and suffers in the presence of nonlinear trajectories with multiple overlapping tracks and missing data. These issues can be better handled by the physics-based models. While tracking, they consider the spatio-temporal patterns of the vessels and proved to be more effective for tracking maritime vessels than Kalman filtering [1]. However, this framework uses only the most recent datapoint for track association, thereby ignoring all the previous datapoints and the information hidden in them. These sequences often contain the long-term trajectory pattern of vessels and can help in future track association.

Deep learning architectures are proven to be quite effective in modeling nonlinear data and thus can also be utilized for trajectory modeling. Recently, deep learning algorithms have been proposed to automatically learn hierarchical feature representations using raw spatio-temporal data quite similar to our case. Architectures such as convolutional neural networks (CNNs) and recurrent neural networks (RNNs) can capture spatial and temporal correlations present in the data. RNN is preferred to model any data that exhibit dynamic behavior, while CNN has proven to be an ideal approach for extracting spatial features from data [15]. The LSTM has been previously used in trajectory prediction [4]. However, the presence of both spatial and temporal features in the vessel trajectories calls for an integration of both of these architectures. With the advancement of our research and computation capabilities, these joint architectures are evolving every year and more sophisticated frameworks are now taking place [16]. In light of these developments, we propose an integrated CNN-LSTM framework in this study. The academic contribution of our study is summarized as follows:

■ To enable the effective tracking of marine vessels, we design and develop a robust integrated 1D CNN-LSTM framework which comprises two interdependent component architectures that work in tandem to achieve accurate tracking results.
■ To capture the essential spatial characteristics from the dataset, we meticulously designed and fine-tuned a CNN component architecture.
■ To account for the temporal dynamics and long-term dependencies within the network nodes, we devise and optimize an LSTM component architecture.
■ To determine the optimal number of layers in both the CNN and LSTM components of our architecture, we conducted an ablation study, enabling us to make informed design decisions.
■ Furthermore, our proposed framework offers the flexibility to track any number of vessels effectively. We demonstrate this using a small-scale dataset of 5 vessels and a large-scale dataset of 327 vessels.

Our proposed approach has several strengths. First, it carries out a data preprocessing procedure to deal with issues such as an incomplete dataset and gaps in the data collection. Second, it utilizes a CNN architecture to help the dense LSTM layers in extracting important spatial features from the temporal data. Third, the data are fed into the LSTM architecture, a recurrent neural network that unravels and models the trajectory pattern of each specific vessel under consideration. Finally, the trained model is utilized to associate each incoming observation to its true track in real-time. We also compared the performance of our approach with other deep learning architectures using metrics such as the F1 score, recall, and precision.

The rest of the paper unfolds as follows. Section 2 highlights some of the existing research areas relevant to the track association problem based on the AIS or trajectory data. Section 3 discusses the data format, variables, and characteristics of the training and test datasets. It also describes the proposed methodological framework and network architecture for track association. Section 4 presents the performance metrics utilized and summarizes the comparative performance of our approach. Finally, we conclude the paper in Section 5.

## 2. Literature Review

The process of monitoring an object over a sequence of frames to determine its location and direction is referred to as object tracking [17]. Both single and multi-object tracking techniques have been used in various applications, including computer vision problems, human behavior analysis, and security surveillance to name a few. Multi-object trackers (MOTs) can be particularly useful in the context of marine security and surveillance, as they can combine information from multiple sensors like radar and sonar, instead of solely relying on AIS data and tracking several vessels in real time [1].

However, applying MOT approaches to AIS data for track association is not straightforward and presents several challenging issues. Firstly, these approaches must appropriately handle the ambiguity surrounding the number of vessels. Sometimes, this information is unknown and must be learned on the go. Secondly, object (vessel) appearance and disappearance are often uncertain, as they may emerge at unexpected times and locations and depart at the tracking boundary. Finally, multiple incidences of overlapping tracks and time gaps must be considered during the tracking process [18]. In recent decades, numerous research efforts have been undertaken to develop innovative and efficient solutions to multiple object-tracking problems. However, very few of them can handle all the issues posed by the AIS track association problem. The following subsections summarize these efforts into a few major areas namely sequential tracking models, physics-based models, machine learning-based and hybrid models, and finally deep learning approaches.

### 2.1. Sequential Tracking Models

Sequential tracking algorithms, such as global nearest neighbor (GNN) [12] and joint probabilistic data association (JPDA), [13], are commonly employed to update tracks based on the contribution of several objects. These algorithms utilize a cost assignment matrix to minimize costs and employ soft assignment, also known as track association probability, to achieve this goal. While global nearest neighbor (GNN) [12] and joint probabilistic data association (JPDA) [13] focus on a single hypothesis for tracking objects, there are other techniques available. For example, multiple hypotheses tracking (MHT) [14] constructs a tree of hypotheses for each item and computes the likelihood of each track to determine the most probable combination of tracks. The random finite set (RFS)-based approaches were also been utilized for tracking objects, as they are capable of handling the inherent uncertainty involved in the tracking process [18]. The majority of sequential tracking-based algorithms are based on the Kalman filtering (KF) approach [19] or its variations, which are frequently employed to track moving objects and provide information regarding their velocity and acceleration based on their position. However, the accuracy of KF is predicated on the assumption of linear motion, and it struggles to accommodate nonlinear motion patterns. Furthermore, the KF framework has limited capacity for handling the distinct characteristics of vessel movements.

### 2.2. Physics-Based Models

The physics-based approaches rely on mathematical equations to describe the motion of ships, taking into account factors such as mass, force, and inertia. These equations utilize physical laws to calculate the future motion characteristics of the ship [20,21,22,23]. Such motion models can be useful for developing simulation systems to study ideal ship kinematic characteristics or even to train navigation systems. However, applying these models to track the trajectory patterns of multiple ships can be challenging. While these models can incorporate the spatio-temporal patterns of vessel movements [1] in the learning process, they are still limited in nature, only considering the last known position to track vessels.

### 2.3. Machine Learning and Hybrid Models

These methods rely solely on historical data and employ machine learning techniques to learn from past information, enabling them to predict future positions when provided with a new feature vector. These prominent machine learning methods used in trajectory prediction studies include the Gaussian process, support vector machine, principal component analysis (PCA), etc. While these methods [24,25,26,27,28] typically perform well in predicting immediate future positions, their prediction accuracy tends to decrease as the prediction time span increases. Furthermore, the performance of these models is highly dependent on the proper tuning of hyperparameters, which can be difficult to achieve. Additionally, they are not capable of processing long sequences and unraveling the spatial and temporal dependencies present in sequential observations. Hybrid approaches, on the other hand, combine physics-based models and machine learning models or different machine learning models to enhance the quality of the trajectory tracking process [29,30,31,32,33,34]. These approaches, however, are not free from the limitations imposed by the physics-based and machine learning models.

### 2.4. Deep Learning-Based Models

Deep learning, which is a subclass of machine learning models, stands out from the rest due to its superior learning capabilities. In the context of marine vessel trajectories, neural networks have been widely used for their ability to process large datasets and discover long-term patterns hidden in vessel trajectories.Because of the robust adaptability, the earliest form of neural network, including the multi-layer perceptron (MLP) [35] and artificial neural network (ANN) [36,37] played a significant role in traffic and marine vessel trajectory prediction. Nevertheless, despite their wide applications, these neural networks exhibit low interpretability. Additionally, they present substantial challenges in terms of spatial and temporal information processing capability [38] since these networks are not equipped to handle such characteristics.

The exploration of incorporating sequential temporal patterns into marine ship trajectory prediction has motivated researchers to investigate the potential application of recurrent neural networks (RNNs) [19]. However, RNNs encounter challenges in capturing long-term dependencies within a sequence due to the issue of vanishing gradients during backpropagation [39]. As a result, the limited long-term memory of these networks can hinder their performance when the data contain significant long-term dependencies [5,40,41]. Two prominent variations of recurrent neural networks (RNNs), specifically long short-term memory (LSTM) [42,43] and gated recurrent unit (GRU) [44], have garnered substantial attention for their remarkable ability to uncover underlying patterns within extended input sequences, proving particularly advantageous for trajectory prediction.

Further advancements in research have led to the utilization of more efficient variants of LSTM and GRU, such as bidirectional LSTM (Bi-LSTM) [4,45], bidirectional GRU (Bi-GRU) [46], context-aware LSTM (C-LSTM) model [47], and multi-step prediction LSTM (MP-LSTM) [48]. Distinct from traditional LSTM, Bi-LSTM has the ability to process data from both past and future contexts. This bidirectional information processing, encompassing both forward and backward information, empowers Bi-LSTM to capture a comprehensive understanding of the sequence. Consequently, numerous innovative models based on Bi-LSTM have been proposed for ship trajectory prediction. However, these models often exhibit significant computational complexity and limited generalization capabilities. The design parameters of these neural network-based frameworks are adjusted in real-time as the vessel progresses, enabling them to identify all potential trajectories a vessel may follow and reconstruct (predict) its trajectories for future time points [37]. Additionally, LSTM networks have demonstrated remarkable multitasking performance [49].

Another neural network, the convolutional neural network (CNN), originally devised to address computer vision problems, has also been explored for the trajectory prediction and classification of the tracks [49] as it can help capture the spatial patterns exist in the trajectory data. Instead of using the original features, several methods advocate the use of latent features derived from the neural network architecture. These methods leverage latent space representation using variational recurrent autoencoder (VRAE) [50] or LSTM [51]. These latent features can capture the spatial patterns present in the data. Temporal ordering and attention maps are also proven to be effective for object tracking [52].

Instead of using the original features, several methods advocate the use of latent features derived from the neural network architecture. These methods leverage latent space representation using variational recurrent autoencoder (VRAE) [50] or LSTM [51]. These latent features can capture the spatial patterns present in the data. Temporal ordering and attention maps are also proven to be effective for object tracking [52].

In addition to conventional deep learning approaches, the research field has expanded to include the application of hybrid deep learning architectures directly to raw datasets. This advancement goes beyond transforming data into a latent space and aims to reveal both temporal and spatial relationships among features. It has proven to be particularly effective in extracting spatio-temporal relationships within the AIS dataset. Hybrid deep learning-based models for ship trajectory prediction, such as the integration of bidirectional LSTM and RNN (BLSTM-RNN) [53] and CNN-LSTM-SE [54], have emerged as notable techniques due to their rapid learning and adaptability capabilities. These approaches excel in producing highly accurate results when dealing with complex and dynamic trajectory data.

However, it is important to note that these methods primarily focus on predicting the next points by considering the sequence of vessel nodes. This differs from track association, which aims to link vessels to their respective tracks. Furthermore, following the prediction route would require a separate prediction model for each vessel, which can complicate the tracking process when dealing with more than ten vessels. The proposed 1D CNN-LSTM model can overcome all these issues and classify multiple vessels by capturing the spatial and temporal patterns hidden in the data.

## 3. Methodology

This section presents our proposed methodological framework for the track association problem using a 1D CNN-LSTM approach. We discuss the framework’s chronological steps in detail. The section begins by describing the AIS data, followed by an explanation of the data preprocessing techniques used to prepare it for our tracking algorithm. We then provide a summary of the traditional CNN and LSTM architectures, which are the component neural networks of our framework. Finally, we introduce the 1D CNN-LSTM architecture used in this study.

**Figure 1:**
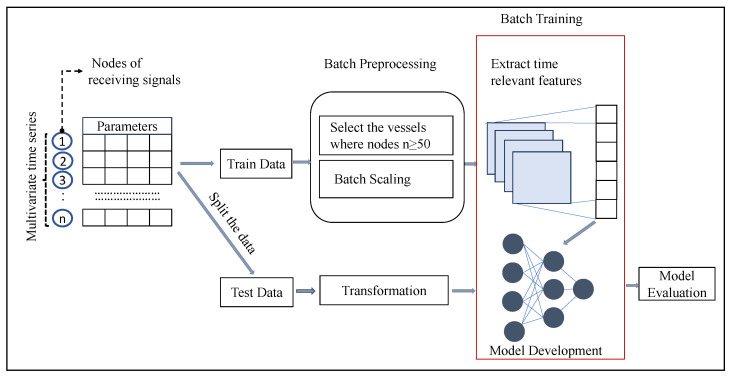
Methodological framework for the track association problem.

The methodological framework depicted in Figure 1 begins by taking the multivariate time series data as input. These data are then split into a training dataset and a testing dataset following a data cleaning procedure. The training data undergo a batch preprocessing and scaling step, while the testing data go through a similar transformation process as the training data. The preprocessed data are then passed through a deep learning layer, which is responsible for extracting relevant and time-dependent salient features. These extracted features are subsequently processed through the next part of the model, which represents the core of the framework. For evaluation purposes, the transformed testing data are utilized to assess the performance of the model.

### 3.1. Data Description

This subsection introduces the automatic identification system (AIS) datasets which will be utilized to develop and test our proposed framework. The AIS dataset was procured from the National Automatic Identification System, maintained by the Coast Guard department of a specific country. When a signal is received in the AIS system, the incoming data are assigned a unique object ID and then verified by a monitoring officer to determine whether the vessel ID is already present in the existing database. The vessel ID, which is a combination of alphanumeric characters, serves as a unique identifier for each vessel in the system. If the received information matches a previously recorded VID, it is retained under the same identifier. However, if the vessel cannot be identified, it is assigned an identifier based on the timestamp. The AIS data are time-stamped and contain relevant information such as speed, the direction of movement, and the location, which are expressed in terms of longitude and latitude. These timestamps serve as nodes for the track association problem.

The database utilized in this study was formatted as a CSV file, comprising comma-separated values. To develop a track association algorithm, a subset of 327 vessels was chosen from the database, where the time intervals between signals are uneven. The irregular signal intervals present a challenge for traditional track association algorithms. To evaluate competing algorithms, approximately 25,000 data points are used for training, and 5000 vessel IDs are reserved for testing. The VID information for these 5000 test data points is intentionally omitted to evaluate the track association accuracy of the competing algorithms.

The dataset contains seven parameters, including a unique key for the database called object ID. The other parameters are vessel ID, timestamp (including date and time), latitude (expressed in degrees), longitude (expressed in degrees), speed (in tenths of knots), and course of direction (in tenths of degrees), as shown in Table 1. For clarity, Vessel IDs, which are lengthy combinations of alphanumeric characters unique to each vessel, have been replaced with labels such as vessel 1, vessel 2, and so on.

**Figure 2:**
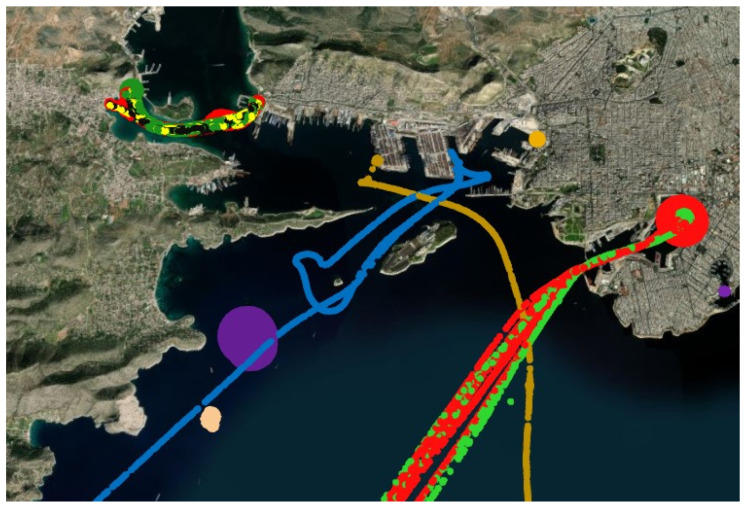
Visualization of vessels and associated tracks in the AIS dataset. Each color corresponds to a track from a different vessel. The map demonstrates the overlapping of the tracks of vessels which makes the track association problem challenging.

For the model development, the timestamps, geographical information in the form of longitude and latitude, and dynamic parameters such as speed over ground and course of direction are selected as the primary input parameters. These selected parameters account for both temporal and spatial information. The vessel ID is designated as the target variable. In Figure 2, the intricacy of the track association problem we sought to investigate is visually demonstrated. The selected vessels exhibit overlapping tracks, creating a challenging scenario that mirrors the complexities present in real-world situations. Each color shown in the figure corresponds to a unique vessel ID. In a subsequent section, the data are preprocessed to ensure that it is suitable for feeding into the proposed model.

### 3.2. Filtering and Data Preprocessing

The AIS data were collected and stored in a large CSV file, featuring data from 327 vessels. The preprocessing procedure begins by splitting the vessels into batches, with each batch comprising approximately 30 vessels. Subsequently, each batch undergoes a preprocessing step, encompassing data cleansing operations. Any timestamp that contains a null value is removed during this stage. Following the completion of the cleaning process, it has been observed that a considerable proportion of vessels lack sufficient data to be chosen for training our model.

**Figure 3:**
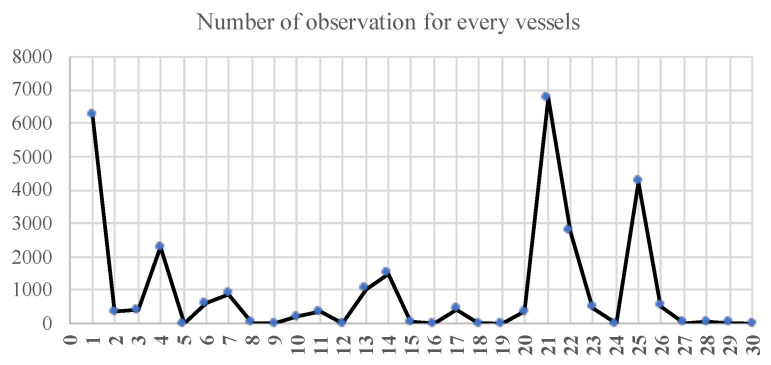
Observation frequency for each of thirty vessels.

Model training parameters are highly dependent on the number of data points available for training. To ensure good accuracy, a threshold of 50 observations (for each vessel) is set to ensure that the model received sufficient data for training. The data distribution for batch 1 is illustrated in Figure 3. Initially, before the preprocessing, it is supposed to contain 30 vessels for training. However, 23 vessels from this batch are eventually used as 7 of them do not meet the data point threshold. In total, 63 vessels out of 327 vessels are excluded from further analysis and we proceed with the remaining 264 vessels, divided into 11 batches, for the model training.

The batch data are then normalized using the standard scaler equation provided below:

(1) z=(X−u)/s

Here, *X* represents the input data, *u* is mean, and *s* represents the unit variance.

### 3.3. Deep Learning Architecture

#### 3.3.1. Convolutional Neural Network (CNN)

Convolutional neural networks (CNNs) are a type of neural network that is designed to process and analyze data with spatial relationships. CNN has been widely utilized in various applications, such as audio signal pattern recognition, image processing, natural language processing, and time series prediction. The CNN architecture proposed by Lecun et al. [55] consists of an input layer, an output layer, and multiple hidden layers. These hidden layers contain convolutional layers, which perform dot products between the input matrix and the convolution kernel [56].

We incorporate the CNN layer as the first layer of our hybrid deep learning architecture due to its ability to extract short-term patterns and dependencies among multiple input variables. They are particularly effective at extracting spatial patterns using convolutional layers, which apply a set of learnable filters to the input data to detect features at different spatial locations. The convolution layer can be multi-dimensional, with its width *d* and height *I* representing the filter dimension. The output of the *t*-th filter, which operates on the input matrix *X*, is calculated below

(2) $$k_{t}=\tanh\left(a_{t}X+b_{t}\right)$$

where *X* is the input matrix, $b_{t}$ is the bias matrix, $a_{t}$ represents the weight matrix and $k_{t}$ is the output function. The output of the filter is then fed into the LSTM layer which is described in the following subsection.

#### 3.3.2. Long Short-Term Memory (LSTM)

Long short-term memory (LSTM) is a variant of recurrent neural network (RNN) [57]. RNNs are a type of artificial neural network that can process sequential data by maintaining a memory of past inputs. In contrast to regular feedforward neural networks that process data in a single pass, RNNs are designed to handle data that have temporal dependencies, such as time series or sequences of text. One of the challenges faced by conventional RNNs is the vanishing gradient problem, in which the gradient of the error function can either diminish or explode during backpropagation when it is propagated through multiple time steps.

**Figure 4:**
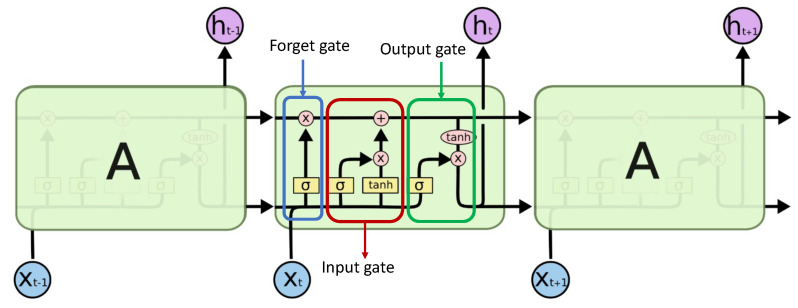
Long short-term memory model schematic diagram.

However, the LSTM architecture overcomes this issue by incorporating a novel memory cell that regulates the information flow through the network. This mechanism selectively retains or discards information, which mitigates the vanishing or exploding gradient problem and allows learning long-term dependencies in sequential data. LSTM enables RNNs to perform long-term sequential prediction [58]. Figure 4 depicts how an LSTM cell operates. The central cell, similar to the other two cells denoted by ‘A’, represents a typical LSTM cell. This cell receives two important inputs: the output sequence generated by the previous LSTM cell and the hidden state value from the preceding cell $h_{t-1}$. Within the cell, there are three gates: a forget gate $f_{t}$, an input gate $i_{t}$, and an output gate $o_{t}$. The forget gate is responsible for determining which information should be discarded from the cell state. Input gate updates the cell state with new information. It has two parts: a sigmoid layer called the “input gate layer” that decides which values to update, and a tanh layer that creates a vector of new candidate values that could be added to the state. The output gate determines the next hidden state. Together, these gates and their respective functions govern the flow of information within the LSTM cell, allowing it to capture and retain relevant information while discarding unnecessary or redundant data. Elaborating on these three gates, the complex mechanism of LSTM architecture is explained below.

In LSTM, the forget gate plays a crucial role in determining which pieces of information to retain or discard from its existing memory, taking into account the arrival of fresh input. Let us denote the input time series as $X=\left(x_{1},x_{2},\ldots ,x_{t}\right)$ and the hidden state of the memory cell as $H=\left(h_{1},h_{2},\ldots ,h_{t}\right)$. The forget gate takes the concatenation of previous hidden state $h_{t-1}$ and the current input $x_{t}$ as inputs, and produces a forget vector $f_{t}$ as output as in Equation (3) [59]:

(3) $$\begin{array}{c} f_{t}=\sigma \left(W_{f}\left[h_{t-1},x_{t}\right]+b_{f}\right) \end{array}$$

Here, σ denotes the sigmoid activation function, which is a nonlinear function that maps its input to a value between 0 and 1. The weight matrix $W_{f}$ represents the weights associated with the forget gate and determines how strongly the inputs affect the forget vector. $b_{f}$ represents the bias vector, which contains constant values that are added to the weighted sum of these inputs and can be thought of as the intercept term in the forget gate equation. The weights and the bias values are learned during the LSTM training process.

LSTM employs the input gate to regulate the inflow of fresh data into the memory cell, which is composed of two components: the input activation gate and the candidate memory cell gate. The input activation gate dictates the extent to which the new input ought to be incorporated into the memory cell, whereas the candidate memory cell gate governs the portion of new data that must be retained in the memory cell.

By taking the previous hidden state $h_{t-1}$ and the current node $x_{t}$ as inputs, the input gate generates an input vector $i_{t}$ and a candidate memory cell vector ${\tilde{c}}_{t}$ in an LSTM. The operation of the input activation gate can be expressed using Equation (4), which involves the weight matrix $W_{i}$ and bias vector $b_{i}$. Meanwhile, Equation (5) illustrates how the candidate memory cell ${\tilde{c}}_{t}$ is formed by applying the hyperbolic tangent activation function (tanh ) to the same set of inputs, using the weight matrix $W_{c}$ and bias vector $b_{c}$.

(4) $$\begin{array}{cc} i_{t} & =\sigma \left(W_{i}\left[h_{t-1},x_{t}\right]+b_{i}\right) \end{array}$$

(5) $$\begin{array}{cc} {\tilde{c}}_{t} & =\tanh\left(W_{c}\left[h_{t-1},x_{t}\right]+b_{c}\right) \end{array}$$

The input vector and the candidate memory cell vector are then combined to update the previous memory cell $c_{t-1}$ (see Equation (6)). Here, ⊙ denotes element-wise multiplication.

(6) $$\begin{array}{cc} c_{t} & =f_{t}⊙c_{t-1}+i_{t}⊙{\tilde{c}}_{t} \end{array}$$

The final gate, the output gate, controls the flow of information from the current memory cell to the current hidden state, which is also the output of the LSTM at the current time step. First, the output vector $o_{t}$ is generated as in Equation (7).

(7) $$\begin{array}{cc} o_{t} & =\sigma \left(W_{o}\left[h_{t-1},x_{t},c_{t}\right]+b_{o}\right) \end{array}$$

Then, the current hidden state, $h_{t}$, is obtained using Equations (8) and (9) where ${\tilde{h}}_{t}$ represents the candidate hidden state value for the current time step of the LSTM network.

(8) $$\begin{array}{cc} {\tilde{h}}_{t} & =\tanh\left(c_{t}\right) \end{array}$$

(9) $$\begin{array}{cc} h_{t} & =o_{t}⊙{\tilde{h}}_{t} \end{array}$$

The following section provides an overview of the hybrid CNN-LSTM model architecture, including a description of the trainable parameters utilized in each layer and the hyperparameters chosen for the model.

#### 3.3.3. One-Dimensional CNN-LSTM Architecture

**Figure 5:**
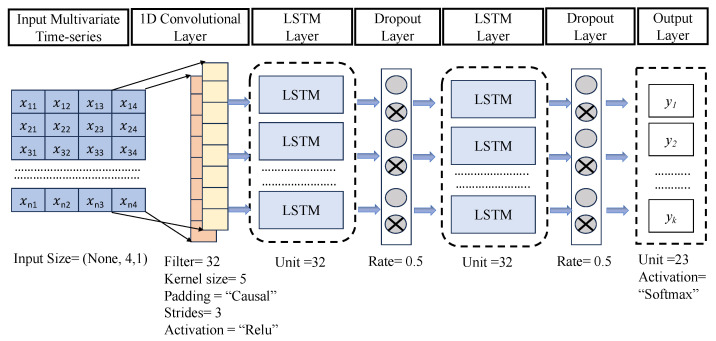
The proposed 1D CNN-LSTM architecture.

To address the track association problem, it is essential to devise a model that can effectively incorporate both temporal and spatial information. The CNN-LSTM model, as illustrated in Figure 5, represents a comprehensive solution that integrates one CNN layer and two LSTM layers to capture the underlying temporal and spatial dependencies present in AIS data. The architecture depicted in the figure showcases the CNN-LSTM model, designed to extract salient features by incorporating an input layer following a 1D convolutional layer. Subsequently, the output from the convolutional layer undergoes further processing through LSTM layers, with the inclusion of two dropout layers after each LSTM layer. The parameter configurations for the CNN, LSTM, and dropout layers are displayed at the bottom of each respective layer in the figure. Finally, the model concludes with an output layer in the form of a dense layer, containing a number of neurons equivalent to the count of vessels present in the training data. The detailed mechanism of the proposed architecture is described below.

**Figure 6:**
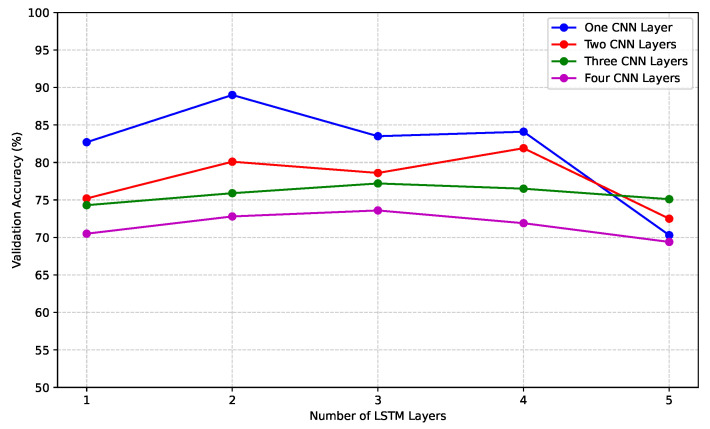
Ablation study for finding the optimized combination of CNN layers and LSTM layers. The combination of one CNN layer and two LSTM layers outperforms the other in terms of validation accuracy score.

The proposed hybrid architecture takes an input vector represented by *X*, where

$$X=\left[\begin{array}{cccc} x_{11} & x_{12} & x_{13} & x_{14}\\ x_{21} & x_{22} & x_{23} & x_{24}\\ \vdots & \vdots & \vdots & \vdots \\ x_{n1} & x_{n2} & x_{n3} & x_{n4} \end{array}\right]$$

has a dimension of (None, 4, 1) and passes it through a convolutional layer. The first dimension, denoted as “None”, signifies the dynamic batch size commonly used in Keras implementation. Keras is a Python-based open-source neural network library that simplifies the prototyping of neural networks. The input vector consists of four variables, expressed as one-dimensional arrays, necessitating the use of a one-dimensional convolutional neural network (1D CNN) in this architecture. The 1D CNN operates with a kernel that can only traverse in a single direction. The proposed deep learning architecture consists of one convolutional neural network (CNN) layer and two long short-term memory (LSTM) layers. This combination has demonstrated a superior performance compared to other CNN-LSTM architectures (see Figure 6). The convolutional layer processes the input and generates an output of size (none, 2, 32) (see Table 2). Here, 32 represents the filter size, while the layer utilizes a kernel size of 5, strides of 3, “Relu” activation function, and “casual” padding. The output volume’s depth determines the number of neurons in a layer connected to the same region of the input volume, with each neuron trained to activate various input features. The stride allocates depth columns around the width and height of the output. The output of the convolutional layer is then passed to an LSTM layer that employs an input, output, and forget gate mechanism. As discussed earlier, the LSTM layer produces an output and a hidden state value.

To avoid overfitting, a dropout layer has been added between two LSTM layers. The dropout technique randomly eliminates certain nodes at a specified probability during each weight update cycle. In this case, a dropout rate of 50% is used, given the large amount of data being trained. The second LSTM layer generates an output with the dimensions (None, 2, 32). The final layer in the architecture is a dense layer that comprises an array of neurons. Each neuron receives inputs from all the neurons in the preceding layer. The output layer utilizes the Softmax activation function to handle categorical variables. Twenty-three neurons are selected for the output layer, corresponding to the twenty-three vessels in each batch. Table 2 lists all the layers and trainable parameters.

To demonstrate the efficacy of the proposed hybrid CNN-LSTM method, extensive experiments are carried out using six distinct methods, including our proposed approach.

Parameters such as the number of filters in the convolutional layer, the size and stride of the convolutional kernel, and the number of hidden cells in the LSTM layer are optimized using the random search method [60] (as specified in Table 3). The optimization of these hyperparameters spans numerous levels, leading to a computationally demanding and time-intensive task. This is due to the vast number of experiments generated by the combination of these hyperparameters.

**Figure 7:**
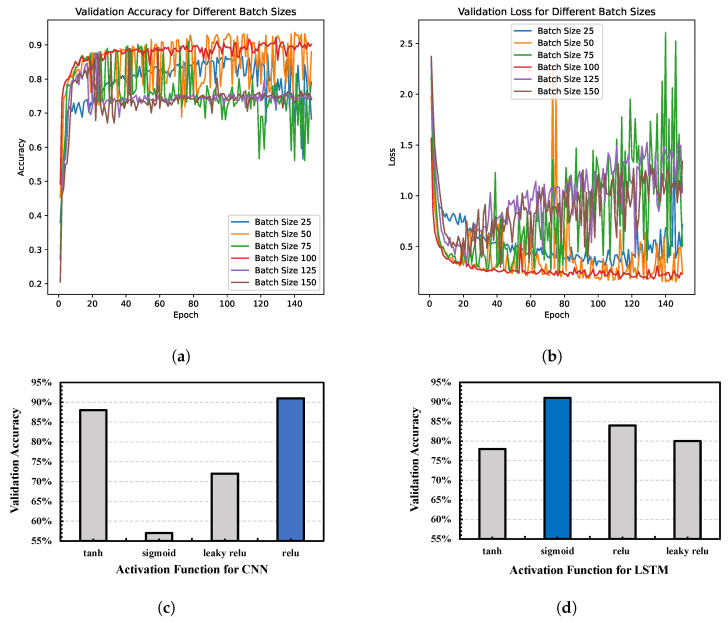
Selecting the optimal hyperparameters using the grid search method. (**a**) Validation accuracy for different batch sizes; (**b**) Validation loss for different batch sizes; (**c**) Performance of different activation functions in CNN layer; (**d**) Performance of different activation functions in LSTM layers.

For the selection of activation functions for the convolutional and LSTM layers, batch size, learning rate, and epochs, the grid search methodology is employed following conventional practices. An ablation study is also conducted to explore the impact of changing hyperparameters, and the results are depicted in Figure 7. It is apparent from Figure 7a,b that a batch size of 100 offers both stability and good performance. Furthermore, it is observed that using an epoch size of 100 yields a satisfactory validation accuracy and validation loss. Although further increasing the epoch size could lead to slight improvements, the incremental gains would be significantly outweighed by the increased execution time. Four activation functions were explored for our proposed model, and among them, “relu” performs better for the CNN layers and “sigmoid” performs better for the LSTM layers (see Figure 7c,d). The optimal hyperparameters selected for model training are outlined below.

To assess the predictive accuracy of the hybrid CNN-LSTM model, we compare it with standalone CNN and LSTM models. All models are trained and tested using the same datasets and experimental settings. The models are implemented in Keras with a Tensorflow 2.0 backend. The training, validation, and testing data are split in a ratio of 70:10:20, respectively. The model training process involves two stages: forward propagation and backward propagation. During forward propagation, the relationships between the four input variables and the target variable (vessel ID) are established. First, the batch size is determined, followed by the initialization of the weight parameters *W* and bias *b*. The hyperparameters are set according to Table 3. The output layer of the network architecture employs the Sigmoid activation function, as shown in Equation (10).

(10) $$f\left(x\right)=\frac{1}{1+e^{-x}}.$$

Here, *x* implies the input value. The backpropagation approach is employed to optimize the relationship between the input and target variable. The weight parameters in the network are adjusted to minimize the discrepancy between the model prediction and the actual target value. The optimization process involves minimizing the loss, specifically the categorical cross-entropy loss function ($L_{CE}$). This function quantifies the difference between the true distribution and the predicted distribution of class labels in a multiclass classification problem. It is defined as the negative log-likelihood of the true class, given the predicted probabilities of all classes [61]. The mathematical expression for the categorical cross-entropy loss function is given as follows [62]:

(11) $$L_{CE}=-\sum_{i=1}^{C}y_{i}\cdot log\left(\hat{y_{i}}\right)$$

where $y_{i}$ is the true label for class *i* and $\hat{y_{i}}$ is the predicted probability for class *i*, and *C* is the total number of classes.

In this study, the Adam optimizer [63] is utilized to adjust the weights and biases during backpropagation. It is a stochastic gradient-based optimization method that adjusts the learning rate dynamically for each parameter, based on the gradient’s historical information. The algorithm calculates adaptive learning rates for each parameter using the gradient’s moving average and second moment.

## 4. Results and Discussion

To evaluate the effectiveness of our model, we generate the confusion matrix, which compares the actual classes with the predicted ones. The diagonal entries of the matrix represent the true positive predictions made by the model. However, relying solely on the confusion matrix is not enough to accurately quantify the performance of the model. Therefore, we compute several commonly used evaluation metrics to fully assess the model’s performance, including:

(12) $$\;\mathrm{Sensitivity}\;=\frac{\mathrm{TP}}{\mathrm{TP}+\mathrm{FN}}$$

(13) $$\;\mathrm{Specificity}\;=\frac{\mathrm{TN}}{\mathrm{TN}+\mathrm{FP}}$$

(14) $$\;F1\;\mathrm{score}\;=\frac{\;\mathrm{Sensitivity}\;+\;\mathrm{Specificity}\;}{2}$$

(15) $$\;\mathrm{Accuracy}\;=\frac{\mathrm{TP}+\mathrm{TN}}{\mathrm{TP}+\mathrm{FP}+\mathrm{FN}+\mathrm{TN}}$$

The abbreviations TP, TN, FP, and FN correspond to true positive, true negative, false positive, and false negative predictions, respectively. Since the dataset has a class imbalance, we calculate the micro-average value for all metrics, which aggregates the contributions of all classes to determine the average metric.

**Figure 8:**
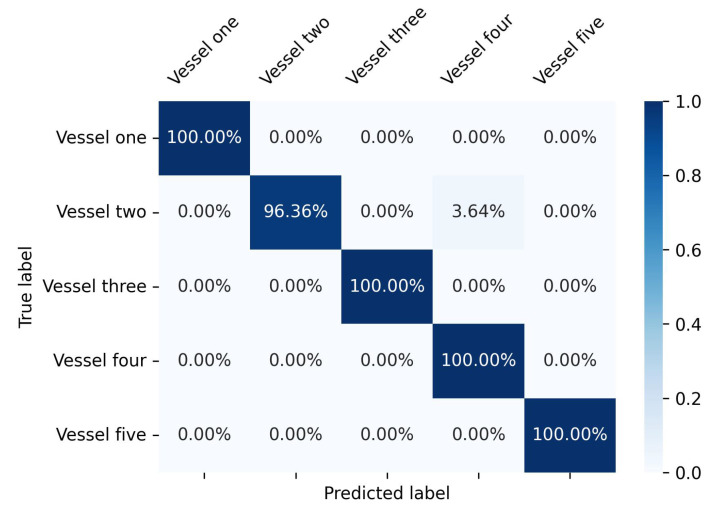
Confusion matrix implementing CNN-LSTM architecture for small scale dataset.

To evaluate the performance of a 1D-CNN LSTM, at first, we start with a small-scale dataset consisting of five vessels. This dataset provides 5688 data points for training and validation, and 1422 data points for testing. The performance of the model is evaluated using a confusion matrix, as shown in Figure 8. The results reveal remarkable accuracy for the small-scale model, with the exception of a single vessel with an accuracy of 96.4%. The exceptional accuracy can be attributed to the diverse routes taken by each vessel, making them easily distinguishable from one another.

**Figure 9:**
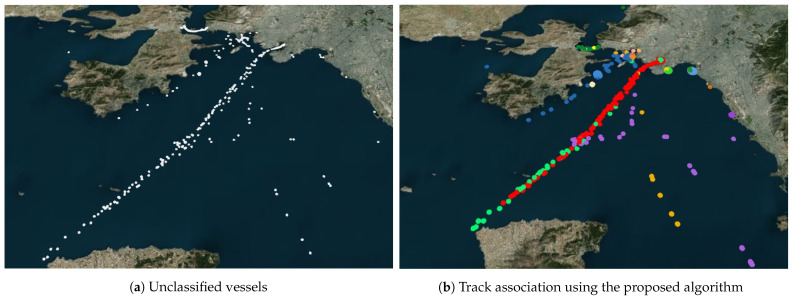
Tracking performance of our algorithm. Thicker white dots in (**a**) represent the unknown objects and (**b**) depicts the assignment of the unknown vessels to their actual track using the proposed algorithm. Different color indicates unique vessels and their tracks.

**Figure 10:**
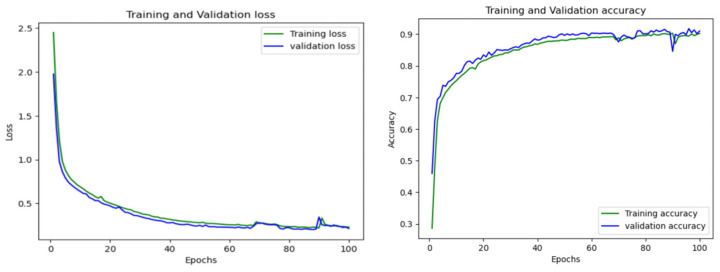
Training and validation loss and accuracy curve for CNN-LSTM architecture.

In order to test the adaptability and versatility of the model, we also conducted a large-scale experiment. This comprehensive evaluation of the 1D-CNN LSTM model is designed to address two important aspects of real-world scenarios: the variation in data size and frequency of data collection among vessels, as well as the presence of overlapping tracks. For the full-scale analysis, all 327 vessels are considered, which span 11 batches, each of which is initially divided into approximately 30 vessels. However, as mentioned in Section 3.2, after the preprocessing, the total number of vessels came down to 264. The performance of the model is evaluated by the average accuracy across 11 batches. This study provides valuable insights into the adaptability of the 1D-CNN LSTM model in complex real-world scenarios and highlights its potential for practical applications. The complexity of the full-scale evaluation of the 1D-CNN LSTM model is illustrated in Figure 9a, where vessel instances without any identifiable information from first batch of vessels are represented by white dots. There are instances of overlapping tracks with no indication of the start and end points. However, as demonstrated in Figure 9b, our model is capable of accurately detecting these vessels and recovering their tracks, even in cases where navigation routes overlap. The exceptional performance of 1D CNN-LSTM is also evident from the stability of its AUC curve for the training and validation loss and accuracy, as shown in Figure 10. The model’s performance can also be quantitatively assessed by comparing its predictions with the true labels.

**Figure 11:**
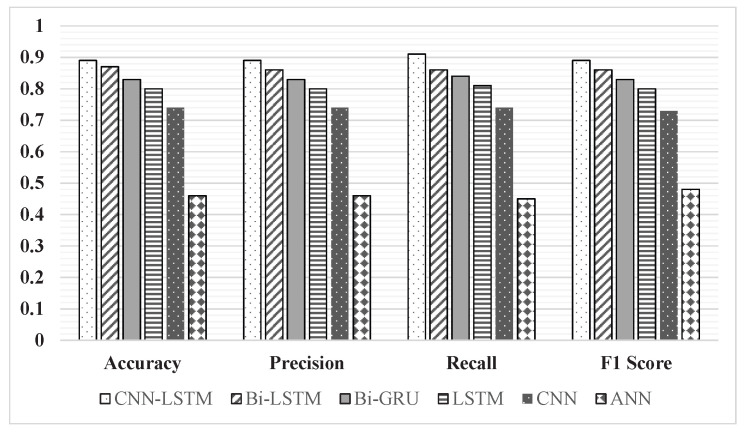
Comparative performance of several deep learning architectures.

Through an extensive quantitative analysis, we evaluated the performance of five distinct deep learning architectures using the same automatic identification system (AIS) dataset. The architectures considered include conventional convolutional neural network (CNN), long short-term memory (LSTM), Bi-directional LSTM (Bi-LSTM) [4,45], bi-directional gated recurrent unit (Bi-GRU) [46], and the feedforward artificial neural network (ANN). We utilize previously detailed performance metrics in this section to assess the models. Remarkably, the results unequivocally showcased the exceptional performance of the 1D CNN-LSTM architecture. Outperforming the CNN, LSTM, Bi-LSTM, Bi-GRU, and ANN models, our proposed architecture achieved an impressive accuracy score of 0.89, a precision of 0.89, recall of 0.91, and an F1 score of 0.89. For a clearer comparative understanding, we also visually depict these results in Figure 11, while detailed metrics can be found in Table 4.

While not the top performer, the LSTM model exhibits a commendable performance, surpassing the CNN model with an accuracy of 80%. Notably, the more complex architectures, Bi-LSTM and Bi-GRU, demonstrate strong competition with accuracy scores of 87% and 83%, respectively. Due to their capacity to learn temporal dynamic behavior and consider the input data in both directions (past and future), these models provide a robust foundation for handling such intricate prediction tasks. However, when compared to the 1D CNN-LSTM model, their performance falls short, highlighting the superior efficacy of the hybrid nature inherent to the 1D CNN-LSTM architecture.

On the contrary, the ANN model showed the weakest performance among all, attaining the lowest accuracy of 46%. This result was anticipated due to the inherent limitations of the ANN model that cannot account for the spatial and temporal patterns inherent in the data, reaffirming the necessity of the judicious selection of architecture based on task-specific requirements.

The superior performance of the 1D CNN-LSTM architecture can be ascribed to its distinctive ability to extract salient features while concurrently considering the sequential nature of the AIS dataset, which is essential for accurate prediction in time series datasets. The architecture takes into account both the spatial and temporal characteristics of the dataset, resulting in exceptional performance in all performance evaluation metrics. Taken together, these findings underscore the efficacy of deep learning models in addressing intricate maritime problems and emphasizing the potential of the 1D CNN-LSTM architecture for future tracking applications.

## 5. Conclusions

Marine vessel track association plays a critical role in national security and surveillance by enabling law enforcement agencies such as the coast guard to detect, monitor, and track potential threat vessels. The availability of large spatiotemporal AIS datasets has provided researchers with the opportunity to develop and evaluate advanced tracking algorithms. In this study, we propose a 1D CNN-LSTM architecture for track association that leverages the strengths of two distinct neural networks to model the spatial and temporal aspects of the AIS dataset. Our numerical study demonstrates that our approach outperforms other deep learning architectures.

However, like other machine learning models, our approach is unable to identify new vessels that may appear during the testing phase. One possible solution is to label these new vessels as anomalies and classify them at the end of the association process. While deep learning models have shown improved accuracy and performance, physics-based heuristics can provide a more comprehensive understanding of the underlying tracks and their dynamics. Therefore, a hybrid model that combines the strengths of both approaches could be a promising avenue for future research.

## Figures and Tables

**Table 1:** AIS information in a CSV format dataset.

ID	VID	SEQUENCE_DTTM	LAT	LON	SPEED	COURSE
1	Vessel 1	2020-02-29T22:00:01Z	37.8567167	23.53735	0	0
2	Vessel 2	2020-02-29T22:00:01Z	37.9483	23.6410167	0	349.9
3	Vessel 3	2020-02-29T22:00:01Z	37.9390233	23.6688483	0	228.3
4	Vessel 4	2020-02-29T22:00:01Z	37.93884	23.6686333	0	0.1
5	Vessel 5	2020-02-29T22:00:02Z	37.9314717	23.6804267	0	170.1
6	Vessel 6	2020-02-29T22:00:02Z	37.9131117	23.5476617	0.1	33.3

**Table 2:** Parameter setting for the hybrid 1D CNN LSTM architecture.

Layer Name	Output Shape	Number of Trainable Parameters
Input layer	(None, 4, 1)	Not applicable
Convolutional layer 1	(None, 2, 32)	192
LSTM layer 1	(None, 2, 32)	8320
Dropout	(None, 2, 32)	0
LSTM layer	(None, 2, 32)	8320
Dropout	(None, 2, 32)	0
Dense layer	(None, 23)	759

**Table 3:** Hyperparameter tuning for the hybrid 1D CNN LSTM architecture.

Parameters	Value
Convolutional layer filters	32
Convolutional kernel size	5
Convolutional kernel stride	3
Convolutional layer activation function	ReLU
Convolutional layer padding	Causal
Number of LSTM hidden cells	32
Number of skip connections	2
LSTM activation function	Sigmoid
Batch size	100
Loss function	Categorical cross entropy
Learning rate	0.0001
Epochs	100

**Table 4:** Evaluation matrix for the deep learning architectures.

Deep Learning Models	Accuracy	Precision	Recall	F1 Score
CNN-LSTM	0.89	0.89	0.91	0.89
Bi-LSTM	0.87	0.86	0.86	0.86
Bi-GRU	0.83	0.83	0.84	0.83
LSTM	0.80	0.80	0.81	0.8
CNN	0.74	0.74	0.74	0.73
ANN	0.46	0.46	0.45	0.48

## Data Availability

Data will be made available upon request.
